# Account of Diseases in an Eastern District from the 20th of November to the 20th of December, 1799

**Published:** 1800-02

**Authors:** 


### STATE OF DISEASES IN LONDON.

*Account of Diſeaſes in an Eaſtern Diſtrict from the 20th of November to the 20th of December,* 1799.

| | No. of Caſes. |
|---|---|
| ACUTE DISEASES. | |
| Typhus Gravior - - | 4 |
| Typhus Mitior - - | 8 |
| Pneumonic Inflammation - | 7 |
| Scarlatina Anginoſa - | 6 |
| Acute Rheumatiſm - - | 4 |
| Hepatitis - - - - | 1 |
| CHRONIC DISEASES. | |
| Catarrh - - - | 15 |
| Cough and Dyſpnœa - | 17 |
| Cough - - - | 10 |
| Hæmoptoe - - - | 1 |
| Phthiſis Pulmonalis - | 4 |
| Pleurodyne - - - | 3 |
| Hydrothorax - - | 2 |
| Dyſpepſia - - | 5 |
| Vomitus - - - | 2 |
| Gaſtrodynia - - | 4 |
| Diarrhœa - - | 10 |
| Dyſenteria - - - | 3 |
| Enterodynia - - | 2 |
| Colica - - - | 2 |
| Scrophula - - - | 2 |
| Icterus - . - | 2 |

| | No. of Caſes. |
|---|---|
| Prurigo - - - - | 3 |
| Dropſy - - - | 4 |
| Anaſarca - - - | 4 |
| Hemiplegia - - | 1 |
| Epilepſy - - - | 1 |
| Nephralgia - - | 1 |
| Dyſuria - - - - | 3 |
| Chloroſis - - - | 5 |
| Hyſteria - - - - | 2 |
| Amenorrhœa - - | 4 |
| Menorrhagia - - | 2 |
| Fluor Albus - - - | 4 |
| Chronic Rheumatiſm - | 15 |
| PUERPERAL DISEASES. | |
| Menorrhagia lochialis - | 4 |
| Dolores poſt partum - | 3 |
| Ephemera - - - - | 4 |
| Maſtrodynia - - | 6 |
| INFANTILE DISEASES. | |
| Meaſles - - - | 5 |
| Scarlatina - - - | 3 |
| Hooping Cough - - | 3 |
| Rachitis - - - - | 2 |

The number of febrile diſeaſes, particularly of the contagious kind, has lately increaſed to a conſiderable degree. The different ſpecies of typhus have prevailed, and, in ſome parts of the town, have proved uncommonly fatal. The ſcarlatina anginoſa has alſo been very general, though not in many inſtances attended with its moſt formidable ſymptoms, nor in any of thoſe referred to in the liſt has it proved fatal.

The very ſtriking change which has taken place in the ſtate of the weather, whilſt it promiſes to leſſen, if not remove the tendency to complaints of an infectious or contagious nature, is likely to produce, or to aggravate diſeaſes of a different claſs. The eaſterly and north-eaſterly winds, which have lately prevailed, have, as uſual, increaſed the number of patients labouring under diſeaſes of the cheſt. The baſtard peripneumony has affected a number of patients in the decline of life, to ſome of whom it threatens to prove fatal; whilſt a hard and dry cough has proved very troubleſome and obſtinate in many caſes of perſons at an earlier period.

*Account*

